# A Comparative Cross-Platform Analysis to Identify Potential Biomarker Genes for Evaluation of Teratozoospermia and Azoospermia

**DOI:** 10.3390/genes13101721

**Published:** 2022-09-25

**Authors:** Suchismita Das, Pokhraj Guha, Monika Nath, Sandipan Das, Surojit Sen, Jagajjit Sahu, Marta Kopanska, Sulagna Dutta, Qazi Mohammad Sajid Jamal, Kavindra Kumar Kesari, Pallav Sengupta, Petr Slama, Shubhadeep Roychoudhury

**Affiliations:** 1 Department of Life Science and Bioinformatics, Assam University, Silchar 788011, India; 2 Department of Zoology, Garhbeta College, Garhbeta 721127, India; 3 Department of Zoology, Mariani College, Mariani 785634, India; 4 GyanArras Academy, Gothapatna, Malipada, Bhubaneswar 751003, India; 5 Department of Pathophysiology, Institute of Medical Sciences, College of Medical Sciences, University of Rzeszow, 35959 Rzeszow, Poland; 6 School of Medical Sciences, Bharath Institute of Higher Education and Research (BIHER), Chennai 600126, India; 7 Department of Health Informatics, College of Public Health and Health Informatics, Qassim University, Al Bukayriyah 52741, Saudi Arabia; 8 Department of Applied Physics, Aalto University, 00076 Espoo, Finland; 9 Physiology Unit, Department of Biomedical Sciences, College of Medicine, Gulf Medical University, Ajman 4184, United Arab Emirates; 10 Laboratory of Animal Immunology and Biotechnology, Department of Animal Morphology, Physiology and Genetics, Faculty of AgriSciences, Mendel University in Brno, Zemedelska 1, 61300 Brno, Czech Republic

**Keywords:** male infertility, teratozoospermia, azoospermia, biomarker genes, *SPA17*, *CCDC90B*, *CCDC91*

## Abstract

Male infertility is a global public health concern. Teratozoospermia is a qualitative anomaly of spermatozoa morphology, contributing significantly to male infertility, whereas azoospermia is the complete absence of spermatozoa in the ejaculate. Thus, there is a serious need for unveiling the common origin and/or connection between both of these diseases, if any. This study aims to identify common potential biomarker genes of these two diseases via an in silico approach using a meta-analysis of microarray data. In this study, a differential expression analysis of genes was performed on four publicly available RNA microarray datasets, two each from teratozoospermia (GSE6872 and GSE6967) and azoospermia (GSE145467 and GSE25518). From the analysis, 118 DEGs were found to be common to teratozoospermia and azoospermia, and, interestingly, sperm autoantigenic protein 17 (*SPA17*) was found to possess the highest fold change value among all the DEGs (9.471), while coiled-coil domain-containing 90B (*CCDC90B*) and coiled-coil domain-containing 91 (*CCDC91*) genes were found to be common among three of analyses, i.e., Network Analyst, ExAtlas, and GEO2R. This observation indicates that *SPA17, CCDC90B,* and *CCDC91* genes might have significant roles to play as potential biomarkers for teratozoospermia and azoospermia. Thus, our study opens a new window of research in this area and can provide an important theoretical basis for the diagnosis and treatment of both these diseases.

## 1. Introduction

The worldwide decline in human semen quality has placed reproductive genetics at the forefront of scientific research on human reproduction and fertility. Male infertility is a combination of complex reproductive ailments with substantial genetic backgrounds [1]. It is characterized by the failure to achieve successful pregnancy after a year of unprotected intercourse [2]. It affects more than 20 million men worldwide [3,4] and the majority of these cases have been diagnosed as idiopathic [5]. Infertile males are characterized by several spermatozoa abnormalities which can be both qualitative and quantitative [6]. Two such diseases which are posing threat to the overall reproductive health of the human populations worldwide are teratozoospermia and azoospermia. Teratozoospermia is one of the emergent qualitative spermatozoa dysfunctions that affect people throughout the world [7] and is defined as the presence of morphologically abnormal spermatozoa in the semen [8]. Sperm morphology is one of the most vital and involute features to define the fertilization capacity of male germ cells [9]. Conversely, azoospermia is a quantitative spermatozoa abnormality represented by the complete absence of spermatozoa in the ejaculate. It is found in approximately 1% of all men and 10% to 15% of infertile males [10]. The etiology of teratozoospermia is closely related to endocrine disorders, environmental factors, life experiences, and molecular defects [11,12]. There are three categorial etiologies of azoospermia, viz, pre-testicular, testicular, and post-testicular [10].

In most cases, the genuine cause of these major types of spermatozoa dysfunctions is unidentified, although some substantial associations have been documented in previous reports [13]. Consequently, the mechanisms leading to these major types of spermatozoa dysfunctions need to be better understood to develop more efficacious treatment strategies. Semen analysis acts as the keystone for identifying spermatozoa abnormalities that lead to male infertility [14]. However, a routine semen analysis can only predict the presence of any abnormality in males and estimate the severity of the problem [14]. Discovering the cause of the abnormality will require epigenetics and deep sequencing studies for the diagnosis of male infertility to identify spermatozoa epigenetic disorders [15,16,17], spermatozoa small noncoding RNA defects [18,19], and other subtle genetic abnormalities that may affect fertilizing potential [20]. Therefore, increasing attention has been given to the function and significance of mRNA in the development and maintenance of spermatozoa. Thus, mRNAs that help to detect spermatozoa abnormalities are potential biomarkers for assessing spermatozoa quality in infertility diagnosis and treatment [21]. Many genes showed a negative association with spermatozoa functioning, for example, aurora kinase C (*AURKC*), spermatogenesis-associated 16 (*SPATA16*), protein interacting with C kinase (*PICK1*), septin 12 (*SEPTIN12*), and nanos C2HC–type zinc finger 1 (*NANOS1*) [22]. In addition, ATP/GTP binding protein like 4 (*AGBL4*) has been found to be upregulated in the spermatozoa of teratozoospermic men [11]. According to Wang et al., the septin 14 (*SEPT14*) gene is predominantly expressed in the testes and neurons [23]. Spermatozoa with *SEPT14* mutations show severe structural defects and high levels of DNA damage [16]. Thus, the identification and analysis of such genes are of great clinical importance for the effective treatment of genetically defective patients and expected therapeutic outcomes for infertile individuals [11]. In an earlier study by Han et al., teratozoospermia datasets were intensively screened using gene set enrichment analysis (GSEA) and weighted correlation network analysis (WGCNA) to find three potential biomarkers, namely, *AGBL4*, *FAM172A*, and *RUNDC3B,* in the teratozoospermia patient group [11]. Another recent study aimed towards finding differentiated genes in the case of patients suffering from azoospermia [24]. However, our work is a pioneering attempt to identify differentially expressed genes that are common to both teratozoospermia and azoospermia, using robust workflow, with the objective to unveil which markers, if any, have a significant role in the process of gametogenesis and spermatozoa development.

Moreover, it is important to find new treatment approaches to avoid time-consuming and painful options, as well as to understand the molecular changes in infertility. Microarray has been widely utilized in recent times to identify candidate biomarkers and therapeutic targets by studying changes in genome-wide gene expressions [25,26]. Some of the circumscribing factors leading to the discordant findings are minute sample sizes, various microarray systems, and different statistical methods. To address these limitations, meta-analysis provides a potent and suitable approach to combining datasets from different studies to improve the reliability and accuracy of findings by increasing statistical power. Gene expression meta-analysis provides incipient biological insights and identifies more precise biomarkers and therapeutic targets [27]. The present study was conducted to identify differentially expressed genes (DEGs) associated with teratozoospermia and azoospermia by performing a meta-analysis on available microarray datasets to understand the common underlying molecular mechanisms. Further, in this clinical condition, we tried to find specific genes to understand the disease mechanism through a protein-protein interaction (PPI) network.

## 2. Materials and Methods

### 2.1. Microarray Data

The National Center for Biotechnology Information-Gene Expression Omnibus (NCBI-GEO) database (http://www.ncbi.nil.nih.gov/geo/, accessed on 1 July 2020) was used to collect suitable gene expression microarray samples [28]. A detailed search was conducted of the GEO database using the individual keywords “teratozoospermia” AND “azoospermia”. Two datasets for teratozoospermia, i.e., GSE6872 and GSE6967, and two datasets for azoospermia, i.e., GSE145467 and GSE25518 were included in our study from the NCBI-GEO database, considering their fulfillment of certain criteria. The datasets which did not show any significant genes in GEO2R were excluded from our study. In the case of teratozoospermia, microarray was used for purified spermatozoa obtained from the ejaculate, while in the case of azoospermia, there is the complete absence of spermatozoa so tissues from testes were used for microarray. Testes tissues contain spermatogonial stem cells (SSCs) which produce spermatozoa through the process of spermatogenesis [29]; therefore, there exists a common origin between the cell type of teratozoospermia and azoospermia. The samples required for the study were collected from both healthy controls and patients. In the teratozoospermia study, controls can be defined as normal fertile males who have fathered at least one child, while in the azoospermia study, males with normal spermatogenesis processes can be considered as controls. The samples for the teratozoospermic study were collected from men aged between 21–57 years while in the case of the azoospermic study, the samples were collected from men of reproductive age. The subjects from whom the samples were collected for teratozoospermic and azoospermic study belonged to the American (from USA and Argentina) and European (from Slovenia) populations, respectively. The gene expression profiling was mainly based on abnormal spermatozoa and tissues of testes. The inclusion criteria that were used while choosing the datasets for meta-analyses are mentioned below:

(i) the sample type should contain RNA only for both “teratozoospermia” and “azoospermia” datasets,

(ii) datasets must not contain intersecting/duplicate data,

(iii) datasets must not be generated from the same research laboratory,

(iv) datasets must be heterogeneous in terms of microarray platform, and

(v) each dataset must contain enough data to carry out a meta-analysis (Table 1). The datasets that matched those inclusion criteria were selected for the present meta-analyses.

### 2.2. DEG Screening and Meta-Analyses

ExAtlas meta-analyses software was used to carry out the analysis of microarray expression data [33]. A total of four GEO datasets were included in the study and the expression profiles of those datasets were extracted using the GEO database. The quantile method was used for the standardization of the data [34]. The datasets were downloaded and saved individually and then merged using the batch normalization method. Gene-specific batch normalization was used to combine two or more datasets. If two datasets included the same tissue or organ, then the median expression levels for the common tissue/organ were neutralized in the two datasets using this method.

ExAtlas and NIA Array Analysis have the same algorithm for statistical analysis [35]. Gene expression values were converted into a log form and used for the analysis of variance (ANOVA) [35], which was modified for multiple hypotheses testing cases. Moreover, the false discovery rate (FDR) [36] was used to evaluate the importance of gene expression changes in place of *p*-values. Thereafter, meta-analyses were carried out based on the saved datasets using the random effect method [37] and lists of DEGs were saved as a gene set file. The random effects method considers the variance of heterogeneity among different studies, which is added to the variance of individual effects. Here the term “effect” means the log ratio of gene expression change/difference compared to the control or study-wide mean or median [38].

The raw datasets were simultaneously analyzed with another software named Network Analyst 3.0 [35]. Upon combining the datasets after their standardization, 15,879 feature numbers were identified and then subjected to batch effect adjustment using Combat [34]. Meta-analyses were then performed on the combined dataset using a random effect model with the *p*-value set to less than 0.05 and FDR to less than or equal to 2. FDR can act as an effective indicator of the strength of a study and the *p*-value can be useful for statistical power analyses. It can also be used to examine thousands of features, such as all the genes of an organism, and measure their expression related to the above-mentioned diseases. The Limma package of R/Bioconductor was utilized for the recognition of DEGs [39].

In addition, gene expression analyses were performed on all the datasets individually using GEO2R [40]. Quantile standardization was carried out and Benjamini and Hochberg’s false discovery rate method [41] was selected by default for GEO2R analysis since it is mostly used for the adjustment of microarray data and also provides a good balance between the discovery of statistically significant genes and limitation of false positives.

### 2.3. Comparative Analyses

A comparison of DEGs from both analyses was carried out and the common genes were marked. The marked genes have an annotation set to the official gene symbol which was then rectified using the db2dbtool of the Biological Database Network (BDN) [42]. In addition, GEO2R was used to generate the gene expression output of all those datasets for comparison [31]. The common DEGs were then marked and also compared with the output of ExAtlas and Network Analyst 3.0. A heatmap was then constructed using the DEGs with the help of the Complex Heatmap Package of R [43].

### 2.4. Protein-Protein Interaction (PPI) Network

DEGs were utilized to carry out the study of PPIs using the STRING application [44]. The protein network file was then opened with Cytoscape software to analyze the core module of the PPI network [11,45]. First (1) shell interactors represent the input proteins that were found to be common between ExAtlas and Network Analyst analyses. No second shell interactors were included in the analysis. The Network Analyzer function of Cytoscape was then used to analyze the generated network. In the network, the nodes represent the proteins, whereas the edges represent the evidence of interactions. The node size is directly proportional to the betweenness centrality value of the particular protein and the node color is based on the degree of connectivity of the different nodes with other neighboring nodes. Nodes with no degree of connectivity were not represented in the network. The difference in the color of nodes was due to their varying degree of connectivity. The highest degree of connectivity was found to be 14, whereas the lowest degree of connectivity was found to be 1.

Furthermore, scatterplots were constructed between the betweenness centrality and closeness centrality of the different nodes and also the betweenness centrality and degree values of the different nodes.

### 2.5. Pathway Enrichment Analyses

The BINGO application of Cytoscape was used to study the biological processes involved with DEGs and functional enrichment analysis [46]. The Benjamini and Hochberg FDR correction was employed to perform a hyper geometric test. For enrichment analyses, the full GO database was selected as the ontology file. The network generated was then analyzed using the network analyzer function of Cytoscape.

**Figure 1:**
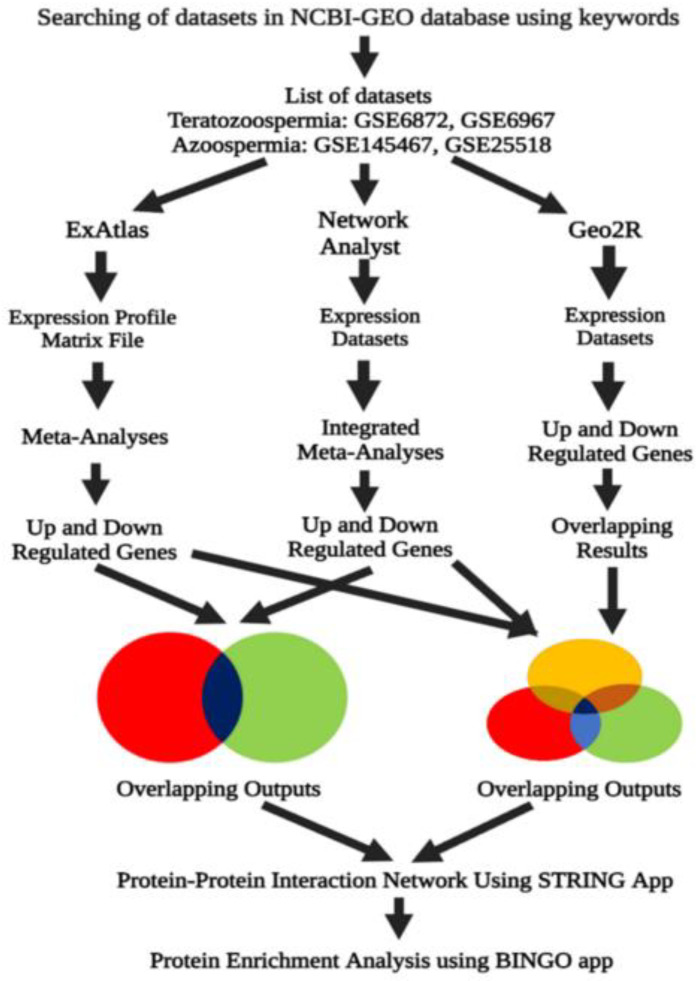
The diagrammatic illustration showing the workflow for the identification of potential biomarker genes common to teratozoospermia and azoospermia, introducing an in silico approach. Meta-analysis using three different analyses, i.e., ExAtlas, Network Analyst, and GEO2R, resulted in overlapping outputs which were then used to study protein-protein interaction (PPI) and protein enrichment analysis using the STRING and BINGO application, respectively.

The overall workflow used in the study for the identification of potential biomarker genes common to teratozoospermia and azoospermia is represented in Figure 1. It is shown that the three analysis methods, i.e., ExAtlas, Network Analyst, and GEO2R were used for the meta-analyses of the genes where overlapping outputs were obtained. These overlapping outputs were then utilized to study the PPI network using the STRING application and pathway enrichment analyses using the BINGO application (Figure 1).

## 3. Results

**Figure 2:**
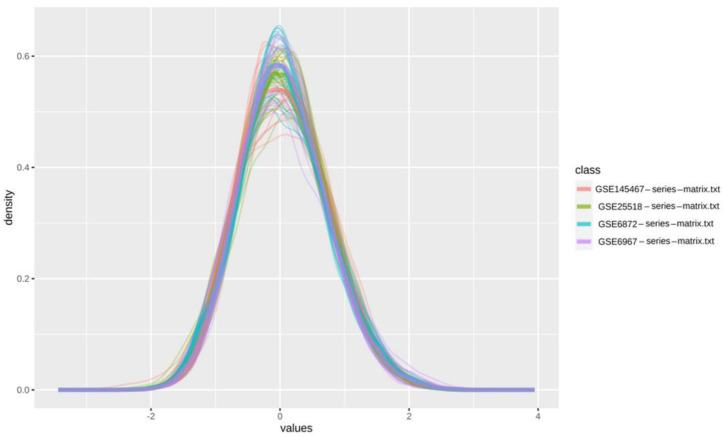
Density plot of the four datasets for teratozoospermia and azoospermia. GSE145467, GSE25518, GSE6872, and GSE6967 have been shown in pink, green, sky, and purple colors, respectively.

Four microarray datasets named GSE6872, GSE6967, GSE145467, and GSE25518 included in the present study (Table 1) altogether consisted of 77 samples, of which, 32 were controls, and the remaining 45 were patient samples. In Figure 2, the distribution of data representing these datasets has been shown with the help of a density plot, which visualizes the distribution of data over a continuous interval or time period, and the peaks of the density plot help to display where the values are concentrated over the interval. In our case, all the curves had their peaks at the interval “0”, meaning that all the values have been concentrated at “0” (Figure 2).

Figure S1 represents box plots constructed using Geo2R which shows the value plots of these four datasets. The plots demonstrated that the log2 values are normalized across all the samples of each dataset with the median line having more or less equal distribution for each dataset.

### 3.1. Expression of Up- and Down-Regulated Genes (i.e., DEGs)

**Figure 3:**
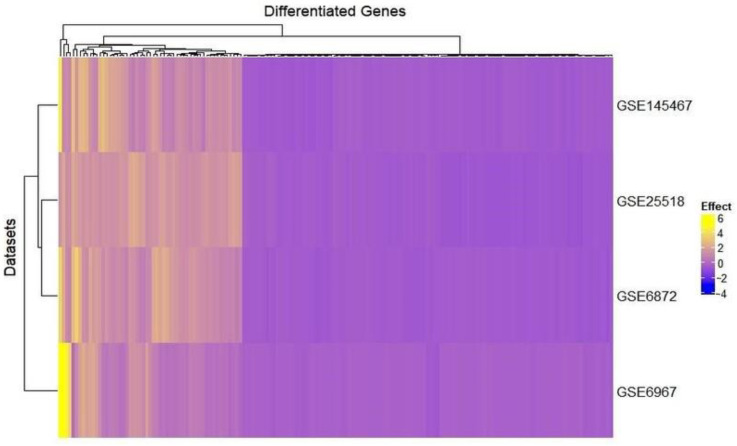
Heatmap of the four datasets of teratozoospermia and azoospermia showing the expression of significantly expressed differential genes as shown by R software using the complete heat package of R. Effect value refers to the change of the log ratio of gene expression compared to the control or study-wide mean or median. Teratozoospermia consisted of the datasets GSE6872 and GSE6967, whereas azoospermia consisted of the datasets GSE145467 and GSE25518.

Meta-analyses of the selected microarray datasets using ExAtlas software revealed 205 significant genes using a random-effect model, of which, 133 were down-regulated and the rest, 72, were up-regulated in the patients compared to healthy controls. Figure 3 represents clustered heatmaps of the four datasets comprising the expression of DEGs. The datasets have been clustered into two groups, namely teratozoospermia and azoospermia, depending upon the expression values of the DEGs. It is clear from Figure 3 that both teratozoospermia and azoospermia follow a similar pattern of gene expression. The effect value in Figure 3 refers to the log ratio of gene expression change/difference compared to the control or study-wide mean or median.

**Figure 4:**
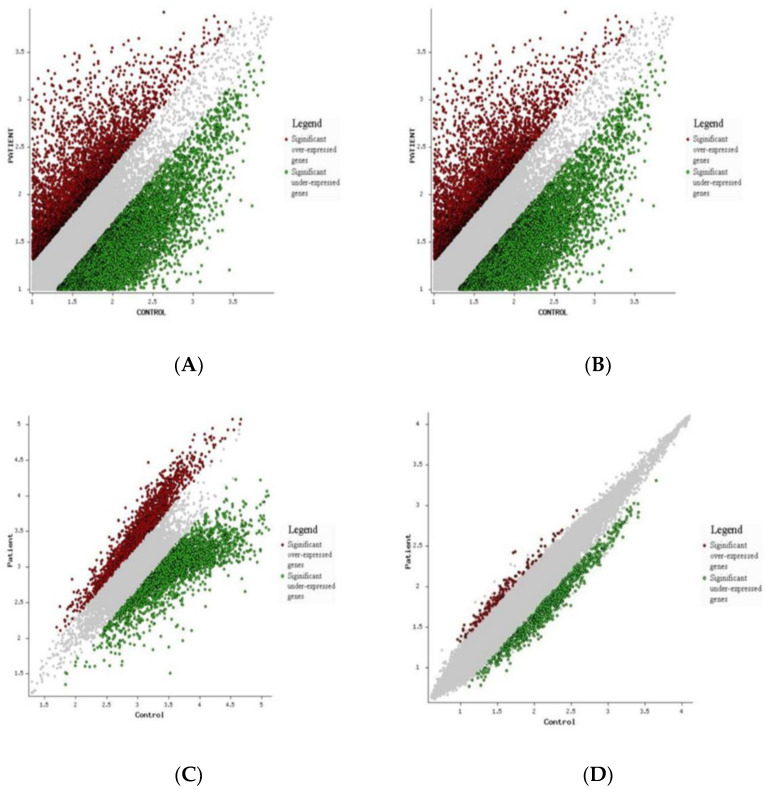
Volcano plots of differentially expressed genes (DEGs) common to teratozoospermia and azoospermia of the four datasets: (**A**) GSE6872, (**B**) GSE6967, (**C**) GSE145467, and (**D**) GSE25518. Significantly over-expressed genes are represented by red dots while significantly under-expressed genes are represented by green dots. Grey dots represent the genes that were not differentially expressed.

The expression pattern across different samples has been shown with the help of Figure S2. Figure 4 represents the volcano plots of significant genes of the four datasets included in our study. A volcano plot is a type of scatterplot that shows statistical significance (*p*-value) versus the magnitude of change (fold change). It also allows for a quick visual identification of genes with large fold changes that are also statistically significant. The red dots in the figure represent significantly over-expressed genes, the green dots represent significantly under-expressed genes, and the grey dots represent the genes that were not differentially expressed (Figure 4).

**Figure 5:**
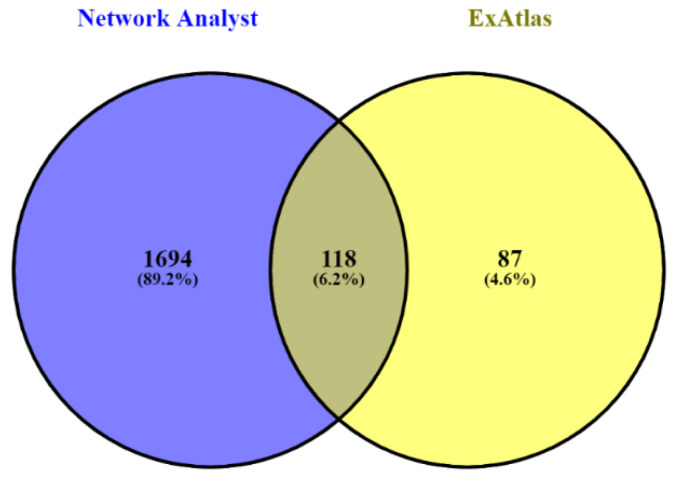
Venn diagram showing the genes common to teratozoospermia and azoospermia found between Network Analyst and ExAtlas. A total of 118 genes have been found to be common in Network Analyst and ExAtlas.

Network Analyst analyses discovered 1812 DEGs, of which, a total of 118 genes have been found to be common when the results of both ExAtlas and Network Analyst were compared (Figure 5). The top 25 DEGs from the above-mentioned 118 genes have been listed in Table 2 based on their fold change (FC) values along with their Entrez ID, log-ratio combined, and FDR value. Surprisingly, among all the DEGs, the sperm autoantigenic protein 17 (*SPA17)* gene has been found to possess the highest fold change value (9.471) from the ExAtlas analysis. This can be considered an important observation since the same gene has been found to have the highest fold change value in the case of Network Analyst analyses. Hence, *SPA17* is negatively expressed in teratozoospermia or azoospermia as it remained down-regulated in the disease conditions as compared to the control. Among these top 25 DEGs, 88% of genes (22) were down-regulated, as apparent from their log-ratio combined value, while the rest 12% (3) were up-regulated (Table 2). Therefore, down-regulated genes were highly expressed as compared to the up-regulated genes.

**Figure 6:**
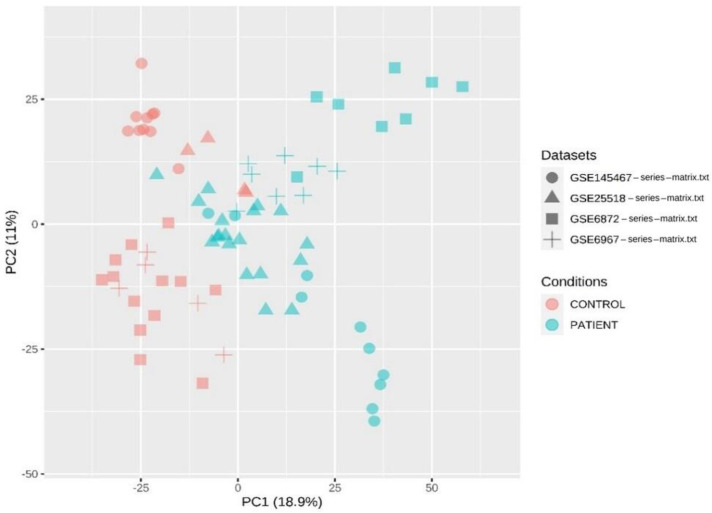
A two-dimensional (2D) PCA plot of the four datasets, two of teratozoospermia and two of azoospermia. In this figure, the round shape represents the dataset GSE145467, the triangular shape represents the dataset GSE25518, the square shape represents the dataset GSE6872, and the “+” symbol represents the dataset GSE6967. The pink color shows the control samples while the sky color shows the patient samples.

Figure 6 shows the two-dimensional (2D) principal component analysis (PCA) of the four datasets, i.e., GSE145467, GSE25518, GSE6872, and GSE6967, two of teratozoospermia and two of azoospermia. This plot shows that similar expression profiles have clustered together. It is considered one of the most famous dimension reduction methods where the information of a complex dataset is converted into the principal component (PC), a few of which can describe most of the variation in the original dataset.

**Figure 7:**
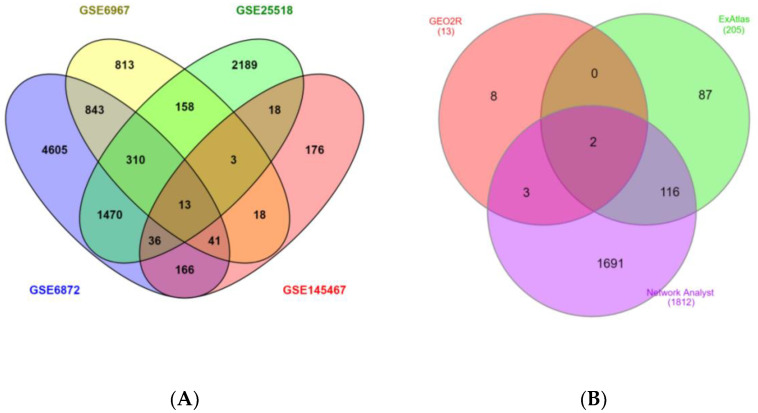
Venn diagrams showing the expression profiles of the study datasets. (**A**) The number of common genes obtained by GEO2R from the two teratozoospermia and two azoospermia datasets as visualized by a Venn diagram; 13 genes were found common to teratozoospermia and azoospermia among the four datasets, (**B**) common genes of individual analyses of the four datasets of teratozoospermia and azoospermia by three different software programs, i.e., ExAtlas, Network Analyst, and GEO2R. Only 2 genes were found common among the individual results obtained across the three analyses, *CCDC90B* and *CCDC91*.

All four GEO datasets were simultaneously analyzed using GEO2R. The expression profiles contained genes that were significantly expressed in comparison to the control. Following this, the expression profiles of all the datasets were overlapped using the Venn diagram (Figure 7A). It has been observed that only 13 significantly over-expressed genes (*p* < 0.05) were common in all four datasets. When these 13 over-expressed genes were compared with the DEGs from ExAtlas and Network Analyst results (Figure 7B), only two genes, *CDC90B* and *CCDC91*, were found to be common to all the three analyses, i.e., ExAtlas, Network Analyst, and GEO2R. The two genes remain down-regulated in the patients having teratozoospermia and azoospermia, as indicated by their negative log ratio combined value. These results shifted our concern towards *CCDC90B* and *CCDC91* genes and prompted our interest in finding the biological function of these as potential biomarkers common to teratozoospermic and azoospermic men, especially in the field of male reproductive health. With respect to Figure 7B, it should be noted that all the genes that have been considered for comparative analyses among the three different software-based approaches (ExAtlas, Network Analyst, and GEO2R) demonstrated a significant fold change in the patient sample compared to the control.

### 3.2. Protein-Protein Interaction (PPI) Network

**Figure 8:**
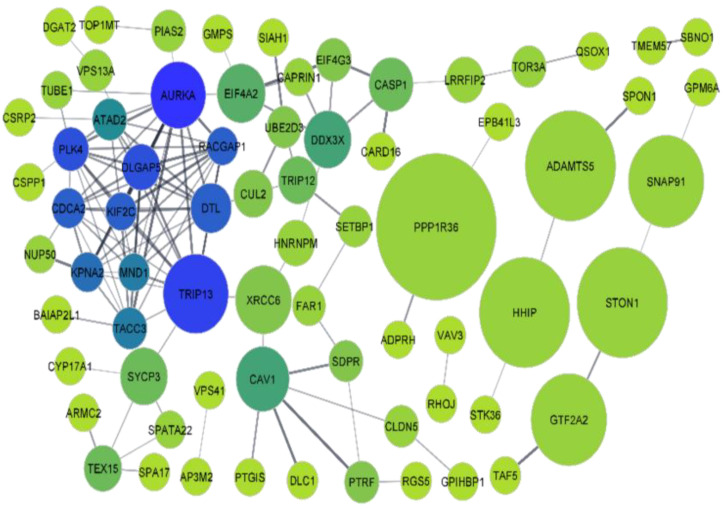
The Protein-Protein Interaction (PPI) network common to teratozoospermia and azoospermia using the STRING application of Cytoscape. The node size is directly proportional to the betweenness centrality value of the particular protein while the node color is based on the degree of connectivity of the different nodes with other neighboring nodes.

Figure 8 represents the PPI network for DEGs. Among 118 first shell interactors or the query proteins, 47 proteins with zero degrees of centrality were not considered during the construction of the network, while the remaining 71 proteins were represented by nodes with different colors. The difference in the color of the nodes is due to their varying degree of connectivity. The blue-coloured nodes represent the protein with the highest degree of connectivity, i.e., 14, while green-coloured nodes represent the proteins with the lowest degree of connectivity, i.e., 1. The transition between the green and blue colors shows the different values of degrees of connectivity between 1 and 14. The node size, on the other hand, is directly proportional to the betweenness centrality value of the particular protein (Figure 8).

**Figure 9:**
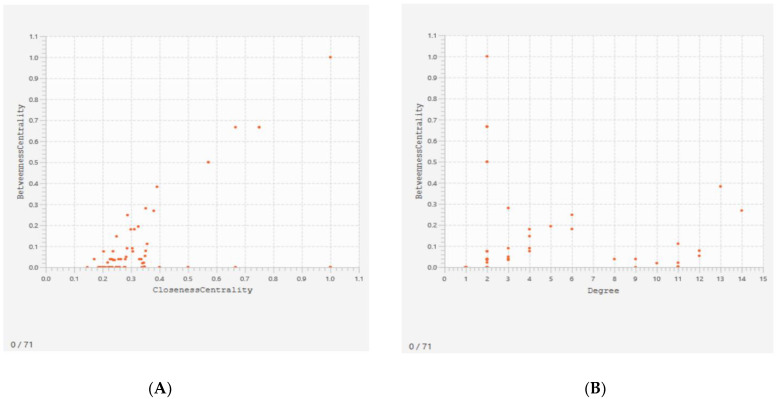
Scatterplots constructed to visualize the position of proteins in the plot with different values of betweenness centrality, closeness centrality, and degree of values. (**A**) Scatterplot between the betweenness centrality and closeness centrality; (**B**) Scatterplot between the betweenness centrality and degree of values.

A scatterplot was constructed between the betweenness centrality and closeness centrality of the different nodes to visualize the proteins having different values. The betweenness centrality of a node is a measure related to the number of shortest paths the node is involved with and the closeness centrality of a node measures its average farness to all other nodes. The scatterplot between the betweenness centrality and closeness centrality showed that PPP1R36 has the highest value of all the proteins (Figure 9A). Another scatterplot was also constructed between the betweenness centrality and degree values of the different nodes where degree represents the number of edges linked to it. In this plot, PPP1R36 has the highest value of betweenness centrality, with a degree value of 2, while the protein AURKA has the highest degree value with a betweenness centrality value of 0.263733 (Figure 9B).

The 20 proteins listed in Table 3 are represented via PPIs without taking secondary interactors into consideration. Table 3 shows the top 20 query nodes, arranged in descending order of their degree of centrality, along with their respective betweenness centrality, closeness centrality, and the average shortest path length. The top 20 proteins in their descending order of degree of connectivity are aurora kinase A (AURKA), thyroid hormone receptor interactor 13 (TRIP13), polo-like kinase 4 (PLK4), disks large-associated protein 5 (DLGAP5), rac GTPase-activating protein 1 (RACGAP1), kinesin-like protein (KIF2C), denticleless E3 ubiquitin protein ligase homolog (DTL), cell division cycle associated 2 (CDCA2), karyopherin α 2 (KPNA2), transforming acidic coiled-coil containing protein 3 (TACC3), meiotic nuclear divisions 1 (MND1), ATPase family AAA domain containing 2 (ATAD2), caveolin 1 (CAV1), dead-box helicase 3 X-linked (DDX3X), eukaryotic translation initiation factor 4A2 (EIF4A2), caspase 1 (CASP1), synaptonemal complex protein 3 (SYCP3), thyroid hormone receptor interactor 12 (TRIP12), testis expressed 15 (TEX15), and serum deprivation-response protein (SDPR). AURKA has topped the list with the highest degree of connectivity (14) followed by TRIP13 and PLK4 with their degree of centrality of 13 and 12, respectively. Centrality can be roughly estimated with the help of the degree of nodes. It can act as an important parameter in a signaling network as it plays a significant role in the estimation of the importance of a node/edge in the flow of information. It also plays an important role in the exploration of drug targets. Among the top three genes having the highest degrees of centrality, TRIP13 (0.383003) has a comparatively higher betweenness centrality value than the remaining two, i.e., AURKA (0.268733) and PLK4 (0.053786). The information flow in a network system can be measured with the help of betweenness centrality. Nodes with a high betweenness centrality can influence the information flow in a biological network which might be helpful as they can act as targets for drug discovery and, hence, are very crucial for network analysis. TRIP13 (0.390977) has the highest value of closeness centrality followed by AURKA (0.379562) and PLK4 (0.348993), respectively. Closeness centrality is another measure that can estimate the rate of flow of information from a given node to another node. On the other hand, TRIP13 (2.557692) has the shortest-path length followed by AURKA (2.634615) and PLK4 (2.865385), respectively. The average shortest-path length measures the accuracy of the information or mass transport occurring on a network. The top 20 interactions from a protein-protein analysis are listed in Table 3.

### 3.3. Pathway Enrichment Analyses

**Figure 10:**
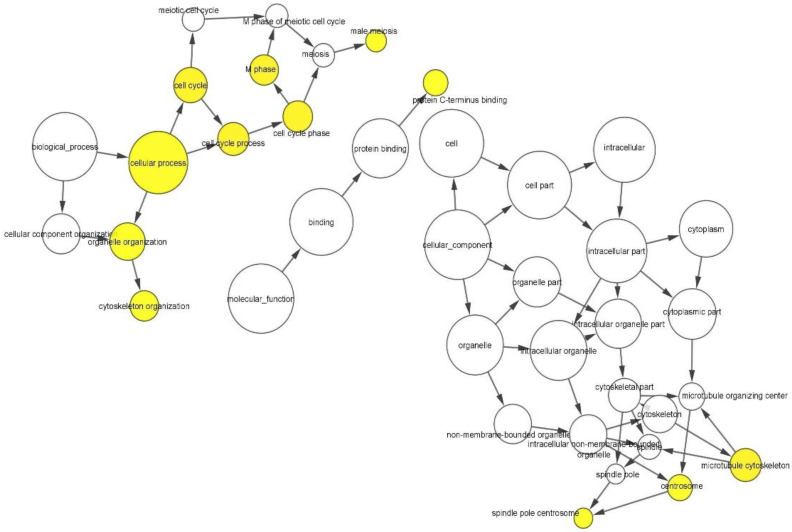
The enrichment network of the shared differentially expressed genes (DEGs) based on the biological process network of DEGs common to teratozoospermia and azoospermia patients using the BINGO application of Cytoscape. Large nodes represent more genes involved and the size of the node is proportional to the number of targets in the GO category. Yellow-coloured nodes are significantly over-represented while the white-coloured nodes are supportive in function.

Gene Ontology (GO) analysis was performed to find out the unique biological significance based on DEGs. In the GO functional enrichment analyses using the BINGO application of Cytoscape (Figure 10), the yellow-coloured nodes have been significantly over-represented while the white-coloured ones are supportive in function. The size of a node is directly proportional to the number of query genes that are annotated to the corresponding GO category.

Table 4 shows the top 12 GO categories based on their respective node sizes which are significantly over-represented in the present study. Among all these significantly over-represented groups, the highest node size has been recorded for cellular processes followed by organelle organization. Neighborhood connectivity has been found to be highest for the microtubule cytoskeleton and centrosome, followed by the cell cycle process.

## 4. Discussion

Male infertility is a reproductive condition with complex etiopathology affecting more than 20 million men worldwide [2,3,4]. In pathozoospermic men, semen abnormalities associated with impaired spermatogenesis manifest as teratozoospermia, asthenozoospermia, oligozoospermia, and azoospermia [47,48]. Azoospermia represents one of the most severe forms of infertility with as high as a 25% risk of genetic disorders [49]. The classical concept of the percentage of morphologically normal spermatozoa below the World Health Organization (WHO)-stipulated lower reference limit [50] also needs to be revisited based on the proposition to include abnormalities in spermatozoa ultrastructure. These spermatozoa abnormalities at the molecular level may explain the underlying mechanism of teratozoospermia, another important male infertility type [51,52]. Several studies have been carried out in recent times for the identification of potential genetic markers in the case of these two spermatozoa abnormalities [11,53,54]. However, a convincing molecular marker common to both teratozoospermia and azoospermia with significant prognostic value has not yet been identified. The ambiguity in identifying the exact biomarkers of these disorders is also attributed to the lack of potential drug targets to improve these infertility conditions.

In an earlier study by Han et al., teratozoospermia datasets were intensively screened to find three potential biomarkers, namely, *AGBL4*, *FAM172A,* and *RUNDC3B,* in the teratozoospermia patient group [11], while another study identified differentiated genes in the case of patients suffering from azoospermia [24]. In this study, most of the datasets shared 25 DEGs, suggesting that they may play a role in the pathophysiology of male infertility. A total of 8 genes (*THEG, SPATA20, ROPN1L, GSTF1, TSSK1B, CABS1, ADAD1,* and *RIMBP3*) were found to be engaged in the overall spermatogenic processes, or at specific stages of spermatogenesis out of the 25 DEGs. They hypothesized that these genes have the potential to be employed as biomarkers for the early diagnosis of non-obstructive azoospermia. However, the potential markers of a single disease cannot unveil the mystery behind the failure of the overall spermatogenic process. Therefore, we made this pioneering attempt to use a rigorous approach to discover differentially expressed genes that are common to both teratozoospermia and azoospermia, with the goal of uncovering certain markers that may play a role in gametogenesis and spermatozoa development and which can be utilized for further downstream processes of identification and subsequent drug discovery.

In the present study, we integrated four datasets, i.e., two for teratozoospermia and two for azoospermia, and successfully identified two genes, *CCDC90B* and *CCDC91,* which are commonly affected in both teratozoospermia and azoospermia. Thus, *CCDC90B* and *CCDC91* might be suitable as common candidate biomarkers in the diagnosis and/or treatment of teratozoospermic as well as azoospermic men.

The genetic basis of pathospermia (including teratozoospermia and azoospermia) has been investigated particularly in relation to the expression of miRNAs [6]. A large number of genes have been found to be associated with pathozoospermia, such as the decrease in spermatozoa concentration; however, fewer genes were found to be associated with abnormalities in spermatozoa morphology, i.e., teratozoospermia [47]. Recent studies on the genetics of teratozoospermia have identified recurrent mutations in three specific phenotypes, macrozoospermia, globozoospermia, and multiple morphological abnormalities of the flagella (MMAF) [47]. Several teratozoospermia-associated gene mutations, including F-box only protein 43 (*FBXO43*) [55], armadillo repeat-containing protein 2 (*ARMC2*) [56], *SEPTIN12* [57], and AGBL carboxypeptidase 5 (*AGBL5*) [58], have been identified by measuring exonic mutations in blood samples using whole exome sequencing technology. Numerous genes that contribute to various sperm abnormalities have recently been discovered through improvements in sequencing methods, particularly in whole exome sequencing (WES). A homozygous loss-of-function mutation in the zinc finger MYND-type containing 15 (*ZMYND15*) gene was found in recent research employing WES. It has been demonstrated that the lack of *ZMYND15* produces nonobstructive azoospermia and severe oligozoospermia [59]; additionally, another research suggests that it may potentially be linked to teratozoospermia [60]. *ZMYND15* has also been described as a switch for haploid gene expression. Proteins such as protein 4.1 [61], spermatogenesis associated 46 (*SPATA46*) [62], cysteine-rich secretory protein 2 (*CRISP2*) [63], spermatogenesis associated 6 (*SPATA6*) [64], and several other genes are believed to play significant roles in the process of spermatogenesis under normal conditions and might act as molecular markers for the clinical diagnosis of pathospermia. The abnormal expression of testicular genes and the loss or mutation of the Y chromosome during spermatogenesis may lead to abnormal sperm morphology, mainly in the expressions of *AURKC*, *SPATA16*, Dpy-19 like 2 (*DPY19L2)*, dynein axonemal heavy chain 1 (*DNAH1),* etc. [65] According to Wang et al., *SEPT14* is predominantly expressed in the testes and neurons [23]. It is the last gene to be identified in the SEPTIN family. Two mutations, A123T and I333T, have been found to be associated with teratozoospermic patients after characterizing the genetic effects of *SEPT14* in cases with abnormal sperm parameters [23]. Spermatozoa with *SEPT14* mutations showed a disruption in the ultrastructure of sperm heads as well as DNA damage. Moreover, the mutation also showed a decrease in the polymerization ability of the spermatozoa [23]. Deleted in azoospermia (*DAZ*) and testis-specific protein Y-linked 1 (*TSPY1*) genes have been found to be expressed at the pre-meiotic stage, whereas transition protein 1 (*TNP1*), protamine 2 (*PRM2*), synaptojanin 2 (*SYNJ2*), and zona pellucida binding protein (*ZPBP*) genes are expressed particularly at the post-meiotic stage [66]. In addition, eight genes, named the testicular haploid expressed gene (*THEG*), spermatogenesis associated 20 (*SPATA20*), rhophilin associated tail protein 1 like (*ROPN1L*), glutathione transferase 1 (*GSTF1*), testis-specific serine kinase 1B (*TSSK1B*), calcium-binding protein, spermatid associated 1 (*CABS1*), adenosine deaminase domain containing 1 (*ADAD1*), and RIMS-binding protein 3 (*RIMBP3*), have been found to be either involved in overall spermatogenic processes or at specific phases of spermatogenesis [24]. In azoospermic conditions, the correlation of the inflammation-associated genes with those essential for spermatogenesis revealed that the genes overlapping in inflammation and spermatogenesis might be used as potential biomarkers for azoospermia [67,68]. Thus, the present study, in relation to the individual previous studies, aims to identify *SPA17*, *PPP1R36, AURKA, TRIP13, PLK4*, *CCDC90B,* and *CCDC91* genes as important biomarkers of both teratozoospermia- and azoospermia-associated infertility in men. However, *CCDC90B* and *CCDC91* genes were identified as the most notable markers and they might play significant roles in the diagnosis and treatment of these two infertility conditions, paving the way to targeted therapy to cure these forms of male infertility.

There are various gene families that are located on the Y chromosome which have been linked to spermatogenic failure and can lead to both teratozoospermia and azoospermia. However, the connection between the defects in these genes and the ensuing fertility problems are not well understood. Studies in mouse models show that a large number of genes involved in both the repair and monitoring of DNA damage have distinct impacts on gametogenesis during the meiotic transition [69].

Meiotic germ cell loss adds considerably to the relatively low efficiency of human spermatogenesis, according to the findings of an in-depth study on the efficacy of spermatogenesis in humans [70,71]. Investigating the expression of certain genes in humans that are involved in meiotic chromatin dynamics has been proven to be an interesting endeavor. For example, the presence of the mismatch repair gene, muts protein homolog 4 (*MSH4*), in human tissues suggests that the encoded protein may play a part in human meiosis [72]. In eukaryotic cells, *cdc2p* (a cyclin-dependent kinase), or one of its orthologs, acts as a master regulator of both the mitotic and meiotic divisions [73]. The *puf-8* gene (a pumilio-related gene) is responsible for controlling RNA stabilization and translation and encodes a ‘pumilio-like RNA binding protein’. In addition to the other pumilio and FBF (PUF) proteins, the PUF-8 protein is necessary for the maintenance of viable germ cells throughout the development process [74]. Additionally, it performs a non-redundant, partly penetrant function in the testes. Primary spermatocytes that do not express PUF-8 are able to complete the prophase of meiosis I; however, they then leave meiosis, re-enter mitosis, and de-differentiate, producing tumorous germ cells. This finding suggests that PUF-8 is essential for primary spermatocytes to continue progressing along the spermatogenesis pathway after completing meiosis [75]. It has also been explained that an aberrant Y material translocation, including the sex determination region (SRY), to the X chromosome may occur during paternal meiosis. This results in the formation of the 46,XX male chromosomal complement. The presence of the SRY gene does not prevent testicular differentiation, however, spermatogenesis is absent since the long arm of the Y chromosome is missing [76]. These males experience normal sexual development, having no structural abnormalities in external genitalia, but they are more likely to suffer cryptorchidism and hypospadias.

The properties of the relevant chromosomes and the breakpoint sites have a major role in predicting the risk of meiotic imbalance. The typical frequency of paternally generated translocation imbalance at prenatal diagnosis is 12%, and many of these imbalances result in fetal mortality [77,78].

More recently, Han et al. identified the *AGBL4* gene which remains significantly upregulated in the spermatozoa of teratozoospermic patients. In their study, the two datasets taken into consideration were GSE6872 and GSE6967. Three common genes were found to be differentially expressed and the expression changes of these differentially expressed genes were further validated using another dataset named GSE6968 [11]. The *AGBL4* gene encodes an ATP/GTP binding protein [79] which is a metallocarboxypeptidase that principally mediates the deglutamylation of target proteins, catalyzes the deglutamylation of post-translational polyglutamate side chains in proteins (e.g., tubulin), and removes polyglutamate from the carboxyl terminus of target proteins (e.g., MYLK) [80,81]. To be the best of our knowledge, no study has so far reported the association of *AGBL4* with male infertility [82], although results from the differential changes of *AGBL4* gene expression proved the feasibility of this gene as a diagnostic marker of clinical teratozoospermia [11]. In contrast to the findings of Han et al. [11], in this study, seven genes have been identified as biomarkers of teratozoospermia and azoospermia, *SPA17, PPP1R36, AURKA, TRIP13, PLK4, CCDC90B,* and *CCDC91*. However, the *CCDC90B* and *CCDC91* genes emerged as the most prominent markers common to both teratozoospermia and azoospermia as confirmed by all three analyses, i.e., Network Analyst, ExAtlas, and GEO2R. These genes remained down-regulated in the patients having teratozoospermia or azoospermia as their log ratios combined value was found to be negative after the analysis. Since these genes remained down-regulated, the production of their products, i.e., proteins, would be lower in such patients.

It is quite apparent from Figure 3 that both teratozoospermia and azoospermia share a similar expression patterns of genes, thereby providing a clear idea of some common biomarkers for the two diseases. Figure 3 also indicates that there exists a clear differentiation between the patient and control groups of each dataset in terms of the expression profiles of the genes. The probability that those above-mentioned 205 genes could be considered as significant biomarkers for both teratozoospermia and azoospermia is partly supported by this observation. It is obvious from Table 2 that the *SPA17* gene has the highest fold change value from both the ExAtlas and Network Analyst, which makes the gene a strong candidate for a potential common biomarker for both male infertility conditions teratozoospermia and azoospermia. The *SPA17*gene encodes a protein present at the cell surface and has an N-terminus with a sequence similarity to human cAMP-dependent protein kinase A (PKA) type II α regulatory subunit (RIIa), while the C-terminus has an IQ calmodulin-binding motif. The middle portion of the protein has carbohydrate-binding motifs and plays a significant role in cell-cell adhesion. The protein was initially characterized by its involvement in the binding of spermatozoa to the zona pellucida of the oocyte [83]. Any mutations/changes in the gene would prevent the association of spermatozoa with the oocyte, resulting in the failure of fertilization and ultimately leading to infertility. More recent studies also show its involvement in additional cell-cell adhesion functions such as immune cell migration and metastasis [84]. Additionally, it plays an important role in cell regulation by participating in signaling pathways through its calmodulin-binding site at the C-terminal [85]. Since *SPA17* is down-regulated, as evident from its log ratio combined value, its expression would decrease in teratozoospermic and azoospermic patients. In addition, the positive fold change value of 9.471 shows that its negative expression increases 9.471 times. Moreover, *SPA17* is involved in the cellular process pathway, as shown in Table 4, so any change in its expression will lead to an alteration of the cellular process as well. It is evident from Figure 9A that the *PPP1R36* gene has the highest value of betweenness and closeness centrality, making it an important biomarker for both teratozoospermia and azoospermia. *PPP1R36* is highly expressed in testes compared to other tissues. It is expressed during gonadal development, especially in testes during spermatogenesis. *PPP1R36* is not only expressed in the developing testes during spermatogenesis but is also present in the acrosome of mature spermatozoa, indicating a role of *PPP1R36* in sperm activity, probably through autophagy [86].

In the case of PPI analyses, the top three genes participating in the network were *AURKA, TRIP13,* and *PLK4*, based on their degree of centrality. The protein encoded by the *AURKA* gene is a cell cycle-regulated kinase that is involved in microtubule formation and/or stabilization at the spindle pole during chromosome segregation [87]. The encoded protein is found at the centrosome in interphase cells and the spindle poles in mitosis. This gene might play an important role in tumor development and progression [88]. *TRIP13* encodes a protein named thyroid receptor-interacting protein 13 which plays a key role in chromosome recombination and chromosome structure development during meiosis [89]. It is also required at early steps in the meiotic recombination that lead to non-crossovers pathways. Moreover, the protein also aids in the efficient completion of homologous synapsis by influencing crossover distribution along the chromosomes, affecting both crossovers and non-crossovers pathways [90]. More importantly, the protein is required for the efficient synapsis of the sex chromosomes and sex body formation [91]. The *PLK4* gene encodes a member of the polo family of serine/threonine protein kinases [92]. The protein localizes to centrioles, complex microtubule-based structures found in centrosomes, and regulates centriole duplication during the cell cycle [93]. *CCDC90B* (Coiled-Coil Domain Containing 90B) is a protein-coding gene. Diseases associated with *CCDC90B* include oculoauricular syndrome and osteochondrosis [94]. *CCDC91* (Coiled-Coil Domain Containing 91), a protein-coding gene, is associated with diseases such as ossification of the posterior longitudinal ligament of the spine and diffuse idiopathic skeletal hyperostosis (https://www.genecards.org/cgi-bin/carddisp.pl?gene=CCDC91 accessed on 2 January 2022).

## 5. Conclusions

In conclusion, the present study has identified 118 DEGs common to the four profile datasets (two belonging to both of teratozoospermia and azoospermia) based on ExAtlas and Network Analyst results. A number of DEGs have been found to be common to both teratozoospermia and azoospermia and may have a diagnostic role in both clinical conditions that may lead to infertility. Among all the DEGs, the significant genes are *SPA17*, *CCDC90B,* and *CCDC91*. The 118 DEGs, after comparison with GEO2R software, showed only two genes, *CCDC90B* and *CCDC91,* to be common in the three analyses, i.e., ExAtlas, Network Analyst, and GEO2R. Therefore, it can be said that *CCDC90B* and *CCDC91* genes could be the potential common biomarker candidates in the pathospermic conditions of both teratozoospermia and azoospermia.

The significantly enriched pathways based on the above-mentioned genes are mainly focused on cell cycle and development processes. These observations could significantly improve our understanding of the causes and underlying molecular mechanisms in teratozoospermia and azoospermia. However, further in vivo analysis of these markers is needed to prove their potentiality and establish their effectiveness as potential drug targets.

## Figures and Tables

**Table 1:** List of microarray datasets included in the study obtained from the National Center for Biotechnology Information-Gene Expression Omnibus (NCBI-GEO) database for teratozoospermia and azoospermia.

Sl. No.	GEO Accession	Subject			Sample	Analytical Platform	Patient Type	Ref.
		Patient	Control	Total				
1	GSE6872	8	13	21	Spermatozoa	GPL570 ([HG-U133_Plus_2] Affymetrix Human Genome U133 Plus 2.0 Array)	Teratozoospermia	[30]
2	GSE6967	8	5	13	Spermatozoa	GPL2507 (Sentrix Human-6 Expression BeadChip)	Teratozoospermia	[30]
3	GSE145467	10	10	20	Testis tissue	GPL4133 (Agilent-014850 Whole Human Genome Microarray 4x44K G4112F (Feature Number Version))	Azoospermia	[31]
4	GSE25518	19	4	23	Testis tissue	GPL570 ([HG-U133_Plus_2] Affymetrix Human Genome U133 Plus 2.0 Array)	Azoospermia	[32]

GEO: Gene Expression Omnibus, GPL: GEO Platform, GSE: Genomic Spatial Event.

**Table 2:** The top 25 up-regulated and down-regulated genes that have been found to be common to teratozoospermia and azoospermia using ExAtlas and Network Analyst are listed along with their Entrez ID, log-ratio combined, and fold change value (FDR).

Gene Symbol	Entrez ID	Log Ratio Combined	Fold Change	FDR
SPA17	Sperm autoantigenic protein 17	−0.9764	9.471	0.0366
TKTL1	Transketolase-like 1	−0.9288	8.488	1.01 × 10^−7^
DDX43	DEAD (Asp-Glu-Ala-Asp) box polypeptide 43	−0.8869	7.708	0.014
PRKAA1	Protein kinase, AMP-activated, α 1 catalytic subunit	−0.8464	7.021	0.000101
SPATA22	Spermatogenesis associated 22	−0.8169	6.561	0.028
HNRNPM	Heterogeneous nuclear ribonucleoprotein M	−0.7491	5.612	4.31 × 10^−6^
TPTE	Transmembrane phosphatase with tensin homology	−0.7235	5.291	2.65 × 10^−7^
EIF4A2	Eukaryotic translation initiation factor 4A2	−0.7106	5.136	0.0251
UBE2D3	Ubiquitin-conjugating enzyme E2D 3	−0.6681	4.656	4.65 × 10^−7^
ADAMTS5	ADAM metallopeptidase with thrombospondin type 1 motif 5	0.6675	4.65	0.0446
OSBPL10	Oxysterol binding protein-like 10	−0.6591	4.561	0.001776
EFHC1	EF-hand domain (C-terminal) containing 1	−0.6357	4.322	0.009339
DLGAP5	Discs, large (Drosophila) homolog-associated protein 5	−0.6311	4.276	0.003758
PPP1R36	Protein phosphatase 1 regulatory subunit 36	−0.6296	4.262	0.004044
TAF5	TATA-box binding protein associated factor 5	−0.613	4.102	0.004949
GTF2A2	General transcription factor IIA 2	−0.6102	4.076	1.13 × 10^−6^
PARM1	Prostate androgen-regulated mucin-like protein 1	0.602	3.999	0.000384
REXO5	RNA exonuclease 5	−0.5984	3.967	0.008627
CDCA2	Cell division cycle associated 2	−0.5947	3.933	0.0338
CLDN5	Claudin 5	0.5839	3.836	0.0363
DGAT2	Diacylglycerol O-acyltransferase 2	−0.5793	3.796	0.000383
PLK4	Polo-like kinase 4	−0.5708	3.722	0.0456
RALGPS2	Ral GEF with PH domain and SH3 binding motif 2	−0.5698	3.714	7.50 × 10^−6^
KIF2C	Kinesin family member 2C	−0.56	3.631	3.96 × 10^−6^
RACGAP1	Rac GTPase activating protein 1	−0.5592	3.624	0.0247

FDR: False Discovery Rate.

**Table 3:** List of top 20 interactions common to teratozoospermia and azoospermia from a protein-protein analysis using the STRING application of Cytoscape. The genes are arranged in descending order of their degree of centrality, along with their respective average shortest path length, betweenness centrality, closeness centrality, and the clustering coefficient.

Name	Average Shortest Path Length	Betweenness Centrality	Closeness Centrality	Clustering Coefficient	Degree
AURKA	2.634615	0.268733	0.379562	0.538462	14
TRIP13	2.557692	0.383003	0.390977	0.615385	13
PLK4	2.865385	0.053786	0.348993	0.621212	12
DLGAP5	2.846154	0.078277	0.351351	0.727273	12
RACGAP1	2.884615	0.002863	0.346667	0.872727	11
KIF2C	2.884615	0.002863	0.346667	0.872727	11
DTL	2.807692	0.11128	0.356164	0.745455	11
CDCA2	2.903846	0.021237	0.344371	0.781818	11
KPNA2	2.942308	0.019194	0.339869	0.777778	10
TACC3	3.038462	0.038629	0.329114	0.75	9
MND1	2.961538	1.68 × 10^−4^	0.337662	0.972222	9
ATAD2	2.980769	0.038462	0.335484	0.75	8
CAV1	3.480769	0.248291	0.287293	0.066667	6
DDX3X	3.211538	0.180979	0.311377	0.2	6
EIF4A2	3.076923	0.194051	0.325	0.2	5
CASP1	4.019231	0.147059	0.248804	0.166667	4
SYCP3	3.346154	0.180241	0.298851	0.166667	4
TRIP12	3.5	0.090196	0.285714	0.166667	4
TEX15	4.230769	0.076169	0.236364	0.166667	4
SDPR	4.211538	0.03449	0.237443	0.333333	3

**Table 4:** List of top 12 significantly over-represented GO categories derived from the BINGO analysis output. The list has been prepared on the basis of ascending order of the adjusted *p*-values.

GO ID	Gene Names	Description	Average Shortest Path Length	Betweenness Centrality	Closeness Centrality	Neighborhood Connectivity	Node Size	No. of Genes	Adjusted *p*-Value
7049	*STEAP3, CDCA2, CUL2, SIAH1, TUBE1, MND1, TEX15, AURKA, RACGAP1, SPIN1, TACC3, KIF2C, TRIP13, SYCP3, KPNA2,* and *DLGAP5*	Cell cycle	2.25	0.176768	0.444444	3	8	16	1.57 × 10^−2^
22403	*CDCA2, CUL2, TACC3, KIF2C, MND1, TEX15, TRIP13, SYCP3, KPNA2, DLGAP5,* and *AURKA*	Cell cycle phase	2.333333	0.320707	0.428571	2.666667	6.63325	11	1.57 × 10^−2^
22402	*CDCA2, CUL2, TUBE1, MND1, TEX15, AURKA, RACGAP1, TACC3, KIF2C, TRIP13, SYCP3, KPNA2,* and *DLGAP5*	Cell cycle process	2.083333	0.34596	0.48	3.333333	7.211103	13	1.57 × 10^−2^
279	*CDCA2, TACC3, KIF2C, MND1, TEX15, TRIP13, SYCP3, KPNA2, DLGAP5,* and *AURKA*	M phase	2.916667	0.017677	0.342857	3	6.324555	10	1.57 × 10^−2^
15630	*PLK4, RANBP9, TUBE1, AURKA, RACGAP1, CSPP1, DLC1, VPS41, SPIN1, TACC3, KIF2C, DTL,* and *DLGAP5*	Microtubule cytoskeleton	2.85	0.014912	0.350877	3.666667	7.211103	13	1.57 × 10^−2^
9987	*EIF4A2, STEAP3, SPON1, USPL1, HHIP, UBE2D3, STON1, AREG, BAIAP2L1, CSRP2, TOP1MT, SPIN1, CASP1, QSOX1, KPNA2, SPA17, STK32B, DLGAP5, VAV3, TOR3A, TKTL1, DGAT2, PTGIS, METTL3, VPS13A, ATP1B2, TEX15, PASK, PIAS2, CLDN5CCDC80, HAND2, NUP50, ADPRH, RHOJ, FAR1, KIF2C, RPP25, DTL, GTF2A2, PRKAA1, CDCA2, CUL2, GMPS, TPTE, CYP17A1, AURKA, AP3M2, WNT6, PPP1R2, RACGAP1, STK36, EPB41L3, PLEK2, PLK4, XRCC6, CAV1, SIAH1, FMO1, RANBP9, TUBE1, MND1, SNAP91, HNRNPM, DLC1, MGAT4A, VPS41, TACC3, TRIP12, TAF5, TRIP13, SYCP3,* and *EIF4G3*	Cellular process	2.083333	0.5	0.48	2.75	17.08801	73	3.59 × 10^−2^
5813	*PLK4, TACC3, TUBE1, DTL, DLGAP5,* and *AURKA*	Centrosome	2.6	0.078421	0.384615	3.666667	4.898979	6	4.92 × 10^−2^
31616	*DLGAP5* and *AURKA*	Spindle pole centrosome	3.25	0.005263	0.307692	3	2.828427	2	4.92 × 10^−2^
6996	*VAV3, XRCC6, CDCA2, CAV1, RANBP9, TUBE1, TEX15, SNAP91, AURKA, RACGAP1, DLC1, EPB41L3, RHOJ, TACC3, PLEK2, KIF2C, TAF5, SYCP3,* and *DLGAP5*	Organelle organization	2.666667	0.234848	0.375	2.333333	8.717798	19	4.92 × 10^−2^
7010	*RACGAP1, DLC1, EPB41L3, RHOJ, TACC3, RANBP9, PLEK2, KIF2C, TUBE1,* and *AURKA*	Cytoskeleton organization	3.583333	0	0.27907	3	6.324555	10	4.92 × 10^−2^
7140	*TEX15, TRIP13,* and *SYCP3*	Male meiosis	3.666667	0	0.272727	3	3.464102	3	4.92 × 10^−2^
8022	*XRCC6, RACGAP1, CAV1, DLC1, SIAH1,* and *EFHC1*	Protein C-terminus binding	2	0	0.5	2	4.898979486	3	4.92 × 10^−2^

## Data Availability

Publicly archived datasets analyzed are available at https://www.ncbi.nlm.nih.gov/geo/query/acc.cgi?acc=GSE6872 (accessed on 18 July 2022) and https://www.ncbi.nlm.nih.gov/geo/query/acc.cgi?acc=GSE6967 (for teratozoospermia, accessed on 18 July 2022), and https://www.ncbi.nlm.nih.gov/geo/query/acc.cgi?acc=GSE145467 (accessed on 18 July 2022) and https://www.ncbi.nlm.nih.gov/geo/query/acc.cgi?acc=GSE25518 (for azoospermia, accessed on 18 July 2022).

## Supplementary Materials

The following are available online at https://www.mdpi.com/article/10.3390/genes13101721/s1, Figure S1: Box plots (value distribution) indicating the value plots of the four datasets of teratozoospermia and azoospermia; Figure S2: Expression pattern (heatmap) across the samples of the four datasets of teratozoospermia and azoospermia.
