# Supplementary material for: A Comparative Cross-Platform Analysis to Identify Potential Biomarker Genes for Evaluation of Teratozoospermia and Azoospermia
Source: Genes (Basel). 2022 Sep 25;13(10):1721. doi: 10.3390/genes13101721 (PMC9602071; doi:10.3390/genes13101721)
Supplement: Supplementary file 1 [file genes-13-01721-s001.zip › genes-1848727-supplementary.pdf]

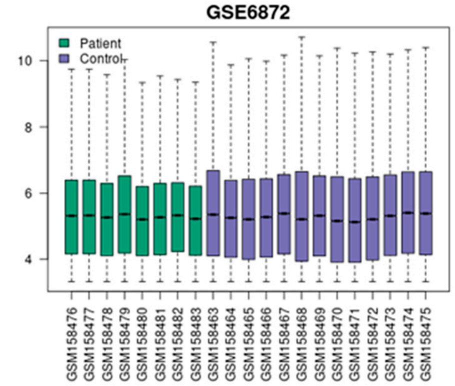

[A]

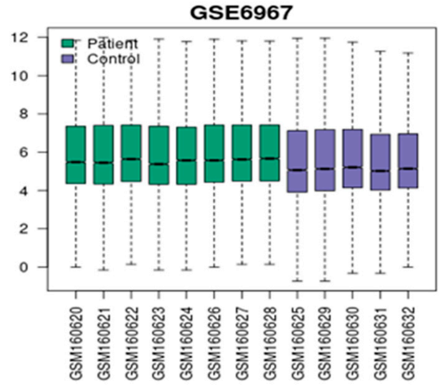

[B]

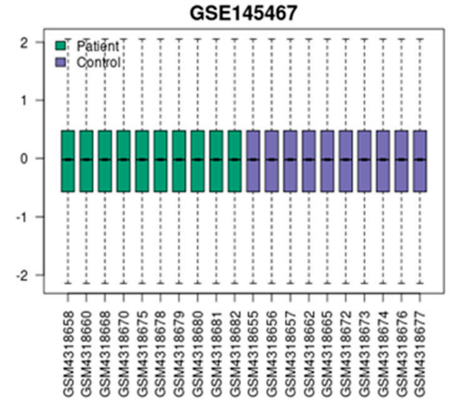

[C]

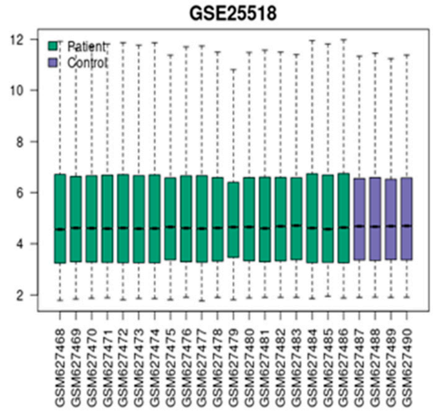

[D]

Figure S1. Box plots (value distribution) indicating the value plots of the four datasets of teratozoospermia and azoospermia. The log2 values are normalized across the samples of each dataset with the median line having more or less equal distribution. Box plots constructed in GEO2R of the two datasets of teratozoospermia (GSE6872 [A] and GSE6967 [B]) and two datasets of azoospermia (GSE14567 [C] and GSE25518 [D]) showing the distribution of expression values of each sample within a dataset. This plot is helpful in determining whether the dataset is normalized, i.e., the value distributions are median centred across samples.

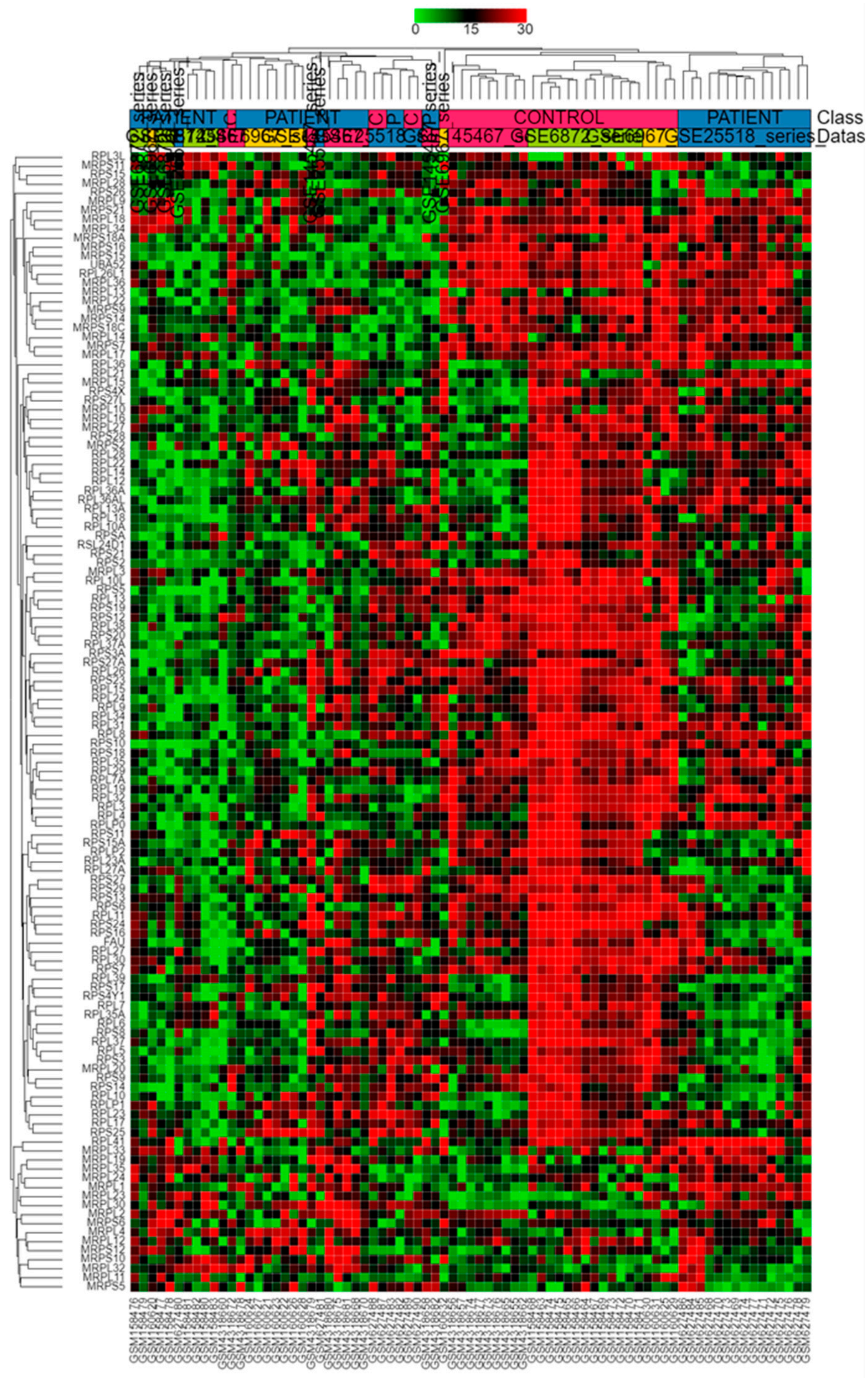

Figure S2. Expression pattern (heatmap) across the samples of the four datasets of teratozoospermia and azoospermia. The horizontal axis represents sample names whereas the left vertical axis presents clusters of differentially expressed genes (DEGs), and the top horizontal axis presents cluster of samples. Green colour represents down-regulated genes while red colour represents up-regulated genes.
